# Genetic variation in the miR‐708 gene and its binding targets in bipolar disorder

**DOI:** 10.1111/bdi.12448

**Published:** 2016-11-16

**Authors:** Alessia Fiorentino, Niamh Louise O'Brien, Sally Isabel Sharp, David Curtis, Nicholas James Bass, Andrew McQuillin

**Affiliations:** ^1^ UCL Molecular Psychiatry LaboratoryDivision of PsychiatryUniversity College LondonLondonUK; ^2^ UCL Institute of OphthalmologyUniversity College LondonLondonUK; ^3^ UCL Genetics InstituteUniversity College LondonLondonUK; ^4^ Centre for PsychiatryBarts and the London School of Medicine and DentistryLondonUK

**Keywords:** bipolar disorder, microRNA, miR‐708, sequencing, susceptibility, variation

## Abstract

**Objective:**

rs12576775 was found to be associated with bipolar disorder (BD) in a genome‐wide association study (GWAS). The GWAS signal implicates genes for the microRNAs miR‐708 and miR‐5579 and the first exon of the Odd Oz/ten‐m homolog 4 gene (*ODZ4*). In the present study, miR‐708, its surrounding region, and its targets were analyzed for potential BD‐associated functional variants.

**Methods:**

The miR‐708 gene and surrounding regions were screened for variation using high‐resolution melting (HRM) analysis in 1099 cases of BD, followed by genotyping of rare variants in an enlarged sample of 2078 subjects with BD, 1303 subjects with schizophrenia, and 1355 healthy controls. Whole‐genome sequencing data from 99 subjects with BD were analyzed for variation in potential miR‐708 binding sites. The minor allele frequencies (MAFs) of these variants were compared with those reported in reference individuals.

**Results:**

Three variants detected by HRM were selected to be genotyped. rs754333774 was detected in three cases of BD, two cases of schizophrenia, and no controls. This variant is located 260 base pairs upstream from miR‐708 and may play a role in controlling the expression of the miR. Four variants were identified in miR‐708 targets binding sites. The MAFs of each of these variants were similar in BD and reference samples.

**Conclusions:**

We report a single recurrent variant located near the miR‐708 gene that may have a role in BD and schizophrenia susceptibility. These findings await replication in independent cohorts, as do functional analyses of the potential consequences of this variant.

Bipolar disorder (BD) is a common disease with a worldwide average population prevalence of 1.4%, which rises to 2.4% if bipolar spectrum disorders are included.^1^ BD is strongly familial, with a 10‐fold increase in risk to the relatives of BD probands.^2^ Estimates of the heritability of BD range from 79% to 93%.^3^, ^4^, ^5^, ^6^ Genome‐wide association studies (GWAS) have identified a number of common polymorphisms which are convincingly associated with BD.^7^, ^8^ The Psychiatric GWAS Consortium Bipolar Disorder (PGC‐BD) working group performed a combined analysis of GWAS data from 7481 individuals with BD and 9250 controls. The same group also tested 34 single‐nucleotide polymorphisms (SNPs), that were associated with *P*‐values of <5 × 10^−5^ in the discovery sample, in an independent replication cohort of 4496 cases with BD and 42 422 controls. The combined analysis of the discovery and replication samples confirmed genome‐wide significant (GWS) evidence of an association between BD and the calcium voltage‐gated channel subunit alpha1C gene (*CACNA1C*), and identified a GWS intronic variant in the Odd Oz/ten‐m homolog 4 gene (*ODZ4*; also known as tenascin‐M4 [*TENM4*]), rs12576775 (*P*=4.4 × 10^−8^).^9^ Both Green et al.^10^ and Mühleisen et al.^11^ later showed further evidence for an association between BD and the same SNP (*P*=6.20 × 10^−9^ and 4.46 × 10^−9^, respectively). A large intergenic region distal to rs12576775 and a proximal genomic region with a high recombination rate in intron 1 of *ODZ4* effectively restrict the BD association region to the genomic interval that includes the first exon of *ODZ4* and the microRNA (miRNA) genes miR‐708 and miR‐5579.^9^ Of the two miRNA genes, only miR‐708 has been experimentally validated and it is known to be expressed in the human nervous system.^12^

The role of miRNAs in psychiatric disorders has recently become prominent as a result of findings in schizophrenia (SCZ). The PGC‐SCZ working group reported GWS with a SNP association in the third intron of the primary transcript of miR‐137.^13^ There is evidence that genetic variation in this gene influences the negative symptoms of SCZ.^14^ In addition to the findings with miR‐137, several other microRNAs have also been implicated in susceptibility to SCZ, including an association with miR‐206 (rs17578796) in a Scandinavian sample^15^ and with miR‐30e (rs112439044) in a Han Chinese sample.^16^ Eight ultra‐rare variants in the precursor or mature microRNA sequences have been reported in American males suffering from SCZ.^17^ An enrichment of rare copy number variations (CNVs) overlapping with microRNAs has also been reported in SCZ, with 25 microRNAs impacted by rare CNVs in two or more unrelated subjects.^18^ Several studies have investigated polymorphic microRNA binding sites in target genes related to SCZ. These include rs3822674, which is located in the 3ʹ untranslated region (UTR) of the gene encoding complexin II (*CPLX2*) and is predicted to interfere with repression of *CPLX2* expression by miR‐498^19^; rs1130354 in the 3ʹUTR of dopamine receptor D2 gene (*DRD2*) was reported to interfere with miR‐326‐mediated repression of *DRD2* expression^20^; and rs11122396 in the 3ʹ UTR of the disrupted‐in‐schizophrenia‐1 (*DISC1*) gene disrupted miR‐135b‐5p‐mediated control of *DISC1* expression.^21^

The evidence that implicates miRNAs in the etiology of BD is not as strong as it is for SCZ. However, recent data have provided increasing support for the hypothesis that miRNAs also play a role in the etiology of BD. In the largest GWAS of BD to date, a SNP in an intergenic region flanking miR‐2113 on chromosome 6q16.1 was the eighth strongest finding.^11^ A gene‐based analysis of all known autosomal microRNAs using the same GWAS data found a significant association between nine microRNAs (including miR‐708) and BD.^22^ In another study, bioinformatic analysis suggested that the BD‐associated glutamate receptor‐7 gene (*GRM7*) 3ʹ UTR variant, rs56173829, might modulate the binding of several microRNAs, including miR‐4295, miR‐130a‐3p, and miR‐130b‐3p.^23^

Postmortem transcriptome studies have shown significant differences between miRNA expression levels in cortical brain tissue from people with BD and controls. While there is substantial heterogeneity in the miRNAs that have been reported to be associated with BD, several miRNAs have been reported in multiple studies.^24^

Two main studies have focused on miRNAs levels in the context of treatment with mood stabilizers in human tissue. One was conducted in lymphoblastoid cell lines, to assess the impact of lithium on miRNA expression.^25^ Of the 13 miRNAs previously suggested to respond to lithium and valproate, the regulation of four (miR‐34a‐5p, miR‐152‐3p, miR‐155‐5p, miR‐221‐3p) was changed after 16 days of treatment. The overall change in miRNA expression was small and no miRNA expression underwent a two‐fold change. A second study focused on miR‐134‐5p. A decrease in miR‐134‐5p plasma levels was observed during manic phases, which correlated with symptom severity. This decrease was reversed upon successful treatment with various mood stabilizers.^26^ Thus, mood stabilizers used for the treatment of BD may influence psychopathology through the modification of miRNA biogenesis.

Bioinformatic analyses indicate that miR‐708 is conserved in mammals within the *ODZ4* intron, suggesting that miR‐708 is co‐expressed with *ODZ4*. The high level of conservation across mammalian species of the miR‐708 precursor stem loop and the guide strand suggests that they are functionally important. It has been demonstrated that the expression of miR‐708 correlates well with the expression of *ODZ4* in adult mouse tissues. The observation of a significant accumulation of miR‐708 and *ODZ4* transcripts in the brain and eyes strongly suggests a physiological role for miR‐708 in tissues in which *ODZ4* is expressed.^27^ A study of postpartum psychosis (a disorder that is prevalent in up to 74% of women with an existing diagnosis of BD and a family history of puerperal psychosis^28^) suggested differential expression of miR‐708 in the monocytes of affected patients compared with controls.^29^ miR‐708 expression has been demonstrated to be upregulated significantly in mouse hippocampal neurons in an oxidative stress cell model.^30^

In the present study, miR‐708, its surrounding region, and its targets were analyzed for possible functional variants associated with BD.

## 1 Materials and Methods

### 1.1 Subjects

The study included 2078 research subjects with bipolar I (BD‐I) or bipolar II disorder and 1303 research subjects with SCZ. The University College London (UCL) control sample included 1355 subjects, comprising 875 screened subjects who had no first‐degree family or personal history of psychiatric illness, and an additional 480 unscreened normal British subjects obtained from the European Collection of Cell Cultures. National Health Service (NHS) multicentre research ethics approval was obtained. All participants provided informed written consent. Ancestry screening was used as a selection criterion for the inclusion of cases. Samples were included if at least three out of four grandparents were English, Irish, Scottish, or Welsh, and if the fourth grandparent was non‐Jewish European, before the EU enlargement in 2004. Participants with BD were interviewed using the lifetime version of the Schizophrenia and Affective Disorder Schedule.^18^ Participants fulfilling the research diagnostic criteria^31^ for BD were included. An analogous protocol was used to recruit participants with SCZ. Blood or saliva samples were collected for all participants. DNA from blood samples was extracted using a standard phenol‐chloroform method and from saliva samples using the Oragene protocol for DNA extraction (DNA Genotek, Ottowa, ON, Canada).

### 1.2 Detection and evaluation of new variants

High‐resolution melting (HRM) variant screening was used to identify BD susceptibility variants 300 base pairs (bp) upstream and downstream from the mir‐708 gene (chr11:79112766‐79113453, GRCh37/hg19).

This method of variant analysis allows cost‐efficient detection of rare variations in large numbers of samples. HRM is particularly amenable to regions not efficiently targeted by established next generation sequencing selection panels such as introns and UTRs.

HRM was performed using three primer pairs in 1099 cases with BD. Reactions were carried out on a LightCycler 480 (Roche, Burgess Hill, UK). Primer sequences and reagents are shown in Table S1. Samples with abnormal HRM curves were then sequenced using the BigDye terminator v3.1 Cycle Sequencing kit (Applied Biosystems, Warrington, UK) on an ABI 3730*xl* DNA Analyzer (Applied Biosystems). Sequencing data were analyzed using the Staden Package (http://staden.sourceforge.net/).^32^ The reference minor allele frequency (MAF) for the general population was evaluated in the data from the 1000 Genomes (1000G) project,^33^ in the Exome Aggregation Consortium (ExAC), Cambridge, MA, USA (http://exac.broadinstitute.org [accessed January 2016]), from the Scripps Wellderly study (n = 534)^34^ and from two population cohorts in the UK10K study (UK10K ALSPAC [Avon Longitudinal Study of Parents and Children] and UK10K TWINS [TwinsUK n = 2432]).^35^ The latter two datasets were queried using the Reference Variant Store, (http://rvs.u.hpc.mssm.edu [accessed 5 August 2016]).

For the ExAC data, it is important to take into consideration the presence of BD and SCZ diagnoses in the database. This did not affect our study because the reported MAFs of the miR‐708 variants selected did not exceed our exclusion threshold for genotyping. Bioinformatic analysis to determine the potential functional effect of SNPs was carried out using the University of California, Santa Cruz genome browser (http://genome.ucsc.edu/), Alibaba 2 (http://www.gene-regulation.com/pub/programs/alibaba2/index.html) and PROMO using version 8.3 of TRANSFACT (http://alggen.lsi.upc.es/cgi-bin/promo_v3/promo/promoinit.cgi?dirDB=TF_8.3).

Only variants with a MAF lower than 0.01 in the general population were considered for further analysis.

### 1.3 Genotyping

Genotyping of the selected SNPs was performed in an enlarged sample of 2078 cases with BD and 1355 ancestrally matched controls. It was performed in‐house using the allele‐specific polymerase chain reaction (PCR), using KASPar reagents (LGC Genomics, Hoddesdon, UK) on a LightCycler 480 real‐time PCR machine. Allele‐specific primers were designed for each of the SNPs using Primer Picker (KBiosciences, LGC Genomics, Hoddesdon, UK; primer sequences are listed in Table S2). The variants were also genotyped in 1305 SCZ samples. For all SNPs, 97% of samples were successfully genotyped. Genotyping for each heterozygote sample was repeated at least twice. All of these data were analyzed to confirm Hardy‐Weinberg equilibrium. Allelic associations for SNPs were performed using Fisher's exact test. Significance values shown for all analyses are uncorrected for multiple testing, and a cut‐off significance value of *P*<.05 was used.

### 1.4 Detection and evaluation of variants in the has‐miR‐708 binding sites

Whole‐genome sequencing (WGS) was performed on 99 of the subjects with BD‐I selected from our BD cohort first on the basis of individuals with a strong positive family history of BD or bipolar spectrum disorder. Where the strength of the family history was tied, individuals with the earliest age at onset were selected. The mean age of onset for the cases selected for sequencing was 21.55 (standard deviation [SD] 8.90), and this was significantly lower than the total cohort (*P* = .0124). The genomic DNA was sequenced using 100 bp paired‐end reads on a Hi‐Seq 1000 (Illumina Inc., San Diego, CA, USA). Sequence data alignment to the National Center for Biotechnology Information human reference genome 37.1 (hg19) and variant calling was performed using the CASAVA 1.8.2 pipeline at Illumina (http://support.illumina.com/sequencing/sequencing_software/casava.html). The sequence data from these individuals was further analyzed and annotated using kGAP (Knome Inc., Boston, MA, USA).^36^ The BD WGS data was screened for variants in miR‐708 binding sites (15 bp upstream and 1 bp downstream the microRNA seed) predicted by Targetscan 6.2: June 2012 (http://www.targetscan.org/).

### 1.5 Imputation and analysis of BD GWAS data

UCL BD and control GWAS data were included in the PGC‐BD dataset.^37^ These data have been subjected to a standardized quality control and imputation pipeline (https://sites.google.com/a/broadinstitute.org/ricopili/). Association testing was performed using PLINK2.^38^

## 2 Results

### 2.1 Variant selection and genotyping

Three single nucleotide variants were detected by HRM analysis across the region selected to be analyzed: rs56158925, rs754333774, and rs768049399 (Table 1). rs56158925 is located 200 bp downstream from mir‐708. This variant has an overall MAF of 0.082 in the general population of the 1000G Project, and the same frequency in the data from the European subpopulation of the project and with a slightly lower frequency in the Wellderly and UK10K cohorts data (Table 1). The other two variants, rs754333774 and rs768049399, are located 260 bp upstream and 27 bp downstream, respectively, of the mir‐708 gene. rs768049399 was annotated in the ExAC database, with an overall MAF of 6.4 × 10^−5^ and 9.7 × 10^−5^ in the European sub population of the project but was not reported in data from the 1000G project, Wellderly subjects or in the UK10K cohorts. rs754333774 was also not reported in the 1000G project or in the UK10K cohorts however it was detected in one of 534 individuals from the Wellderly sample (Table 1). The estimated MAF of this variant in these two datasets is 0.0017.

**Table 1:** SNPs detected by HRM across the region chr11:79112766‐79113453 (GRCh37/hg19); variants: the base change indicated is on the—strand; the genomic reference sequence used is GRCh37/hg19; 1000G, 1000genome project; Eur, European population; Exac, Exome Aggregation Consortium; Wellderly, Scripps Wellderly study; UK10K, UK10K ALSPAC (Avon Longitudinal Study of Parents and Children) and TWINS (TwinsUK); MAF, minor allele frequency; nd, not detected

SNP	Position in ch11	Variants	1000G MAF	Eur 1000G MAF	Exac MAF	Eur Exac MAF	Wellderly MAF	UK10K MAF	Position compared to miR‐708
rs754333774	79113407	G > A	nd	nd	nd	nd	0.000 94	nd	Upstream
rs768049399	79113040	A > C	nd	nd	0.000 064	0.000 097	nd	nd	Downstream
rs56158925	79112878	C > T	0.082	0.082	nd	nd	0.069 29	0.063 54	Downstream

Genotyping assays were designed for the two SNPs with MAFs lower than 0.01 in the general population. Genotyping was conducted in the complete UCL case–control sample, including cases with BD and SCZ. The rs768049399 variant allele was detected in one case with BD and in one control (Table 2). The rs754333774 variant allele was detected in three cases with BD, two cases with SCZ, and no controls (Table 2).

**Table 2:** Genotype counts of microRNA miR‐708 variants in the University College London bipolar disorder (BD) and schizophrenia (SCZ) case–control sample

	Position on ch11	Position compared with miR‐708	Change	N	Genotype counts		MAF
rs754333774	79113407	Upstream	G>A	2041	BD	0/3/2038	0.0007
				1261	SCZ	0/2/1259	0.0008
				3302	BD + SCZ	0/5/3297	0.0007
				1310	CTRL	0/0/1310	0
rs768049399	79113040	Downstream	A>C	2001	BD	0/1/2000	0.0002
				1274	SZ	0/0/1274	0
				3275	BD + SCZ	0/1/3274	0.0001
				1308	CTRL	0/1/1307	0.0004

BD, bipolar disease; CTRL, control; MAF, minor allele frequency; N, total number; SCZ, schizophrenia. The genomic reference sequence used is GRCh37/hg19; *change*: the nucleotide change indicated is on the negative strand; *genotype count*: number of homozygotes for the minor allele/heterozygotes/homozygotes for the major allele.

Bioinformatic transcription factor binding analysis of the effect of rs754333774 did not identify consistent predictions for altered binding. By contrast, the variant allele of rs768049399 was predicted to destroy a binding site for the CCAAT/enhancer‐binding protein beta transcription factor and to create a binding site for five transcriptional factors: hepatocyte nuclear factor (HNF)‐3α; TATA box‐binding protein; homeobox protein D8; HNF‐1C; and HNF1‐B.

### 2.2 Variants in miR‐708 binding sites

Targetscan 6.2 predicted 4377 transcripts with miR‐708 binding sites (including many transcripts with overlapping 3ʹ UTRs), with a total of 381 conserved sites and 4999 poorly conserved sites (including many overlapping sites). These binding sites were analyzed in WGS from 99 subjects with BD for possible variants. Only four nonreference (GRCh37/hg19) allelic variants were identified. The variants were located in the genes encoding the following proteins: apolipoprotein C‐III (*APOC3*); polypeptide N‐acetylgalactosaminyltransferase 13 (*GALNT13*); ST6 (alpha‐N‐acetyl‐neuraminyl‐2,3‐beta‐galactosyl‐1,3)‐N‐acetylgalactosaminide alpha‐2,6‐sialyltransferase 4 (*ST6GALNAC4*); and solute carrier family 22 member 23 (*SLC22A3*) (Table 3). All four variants are known common variants that have been annotated on The Single Nucleotide Polymorphism Database (dbSNP). rs5128 in *APOC3* is 1 bp upstream from the miR‐708 seed sequence, while the other three variants rs707082, rs1043026, rs5873874, located in *GALNT13*,* ST6GALNAC4* and *SLC22A3,* respectively, are within the seed sequence of the miR‐708 binding sites.

**Table 3:** Variants in predicted microRNA miR‐708 binding sites detected by whole‐genome sequencing (WGS) and their frequencies in bipolar disorder WGS (BD WGS) data and in reference data sets

Gene	Seed position in 3ʹ UTR	SNP	Position	Alleles	Ancestral allele	Variant position in the miR‐708 binding site (5ʹ–>3ʹ)	BD WGS	1000G	Eur 1000G	Exac	Eur Exac
*APOC3*	41–47	rs5128	chr11:116703640	G > C	C	..CCUAUCCAUCCUGC**G**agcuccuC..	0.915	0.77	0.88	0.84	0.9
*GALNT13*	764–770	rs707082	chr2:155307832	T > C	C	..AAACUAAACAAUCUUgcucc**u**aG..	0.93	0.76	0.89	nd	nd
*ST6GALNAC4*	359–365	rs1043026	chr9:130670310	A > G	G	..CCUCCCUCCCCAGCCgc**u**ccuaC..	0.995	0.93	1.00	nd	nd
*SLC22A23*	1812–1818	rs5873874	chr6:3271471‐3271471	‐>GAT	GAT	..ACUUGCUCCACGUCCagcuc**‐**cuC..	0.955	0.83	0.96	nd	nd

The genomic reference sequence used was GRCh37/hg19. The variant position in the sequence is indicated with boldened letters.

1000G, 1000 Genome Project; APOC3, apolipoprotein C‐III; Eur, European population; Exac, Exome Aggregation Consortium; GALNT13, polypeptide N‐acetylgalactosaminyltransferase 13; nd, not detected; SNP, single‐nucleotide polymorphism; UTR, untranslated region; SLC22A23, solute carrier family 22 member 23; ST6GALNAC4, ST6 (alpha‐N‐acetyl‐neuraminyl‐2,3‐beta‐galactosyl‐1,3)‐N‐acetylgalactosaminide alpha‐2,6‐sialyltransferase 4.

The allele frequencies of the four variants in the BD WGS samples were similar to those reported in both the entire sample and the European‐only sample from the 1000G project (Table 3). Data were available only for rs5128 in the ExAC database, and these were similar to those of the BD WGS sample. The 3′ UTRs of the remaining genes were not included in the exome sequencing data in the ExAC database. Association testing of GWAS data for the first 491 UCL BD cases and 495 UCL controls, imputed using the 1000G project data as a reference panel, did not show evidence for involvement of rs5128 or rs707082 with BD (*P*=.5214 and .4286, respectively); rs1043026 and rs5873874 were not reliably imputed (Table S3).

## 3 Discussion

The potential role of miRNAs in psychiatric disorders has recently been highlighted by GWS association findings with SNPs within miR‐137 and SCZ.^13^ Several reports have implicated microRNAs in BD, both genetically and biologically.^11^, ^23^, ^25^, ^26^, ^39^, ^40^ The PGC‐BD group performed a combined analysis of GWAS data that identified a GWS intronic variant in *ODZ4* (*TENM4*), rs12576775.^9^ This association has been confirmed by two other independent studies.^10^, ^11^

The two variants in miR‐708 with a MAF lower than 0.01 (rs768049399 and rs754333774) were genotyped in our BD and control cohorts. The strong prior findings with miRNA genes in SCZ led us to genotype the selected variants in our SCZ cohort, in addition to the subjects with BD and control subjects.

The rare allele of rs754333774 was found only in individuals with BD or SCZ; in our own data and, this finding was not statistically significant. This variant was detected in a single individual from the Wellderly sample but was absent in a substantially larger number of individuals from the UK10K cohorts. Together the frequency of this variant in the combined UK10K cohorts and Wellderly sample was lower than that in our BD and SCZ case cohorts. This finding is intriguing but requires validation in large independent samples of subjects with BD and or SCZ and healthy controls. No support for a role for rs768049399 in BD (and/or SCZ) was found.

In order for microRNAs to regulate gene expression, they need to bind to a target region, normally in the 3ʹ UTR of a gene. It is important that nucleotides 2–8 of the miRNA have a perfect match with their target, and this region is defined as a seed region. The miRNA binding site and, more specifically, the seed regions are well conserved; they are more likely to reside within those targets in the transcriptome with lower variant densities, especially target regions in which nucleotides have low mutation frequencies.^41^ Analysis of enrichment of GWS signals for miRNA genes and their putative target regions implicated miR‐137 and its pathway in SCZ in different populations.^42^, ^43^ With the advent of WGS, it is therefore possible to implicate not only regions, but also specific variants, in disease. WGS data for 99 BD subjects were analyzed for possible variants in the miR‐708 binding sites predicted by Targetscan. This analysis identified four miR‐708 binding site variants, each of which was located in brain‐expressed genes, including *APOC3* and *SLC22A23*. Both of these genes have been reported to be associated with the response to antipsychotic drug treatment. Variants in *APOC3* have been implicated in variations of cholesterol and triglyceride levels in SCZ patients treated with clozapine and olanzapine;^44^ variants in *SLC22A23* have been reported to be associated with QT prolongation in a GWAS of subjects with SCZ treated with quetiapine.^45^ However, comparison of the allele frequency data of the four miR‐708 binding site variants between the 99 WGS subjects with BD and those in reference databases suggested that none of the miR708 3 ′UTR binding site variants were likely to be etiological. Indeed, analysis of imputed data from our own BD case–control sample did not show evidence for an association for the two variants that were reliably imputed. One of the challenges of studying miRNAs is the complexity of miRNA‐target gene networks. The study of these networks requires systematic computational prediction of miRNA‐target gene interactions. However, there is a certain degree of imprecision in the predictions made by current miRNA target gene algorithms. This imprecision can be observed in the inconsistent prediction scores that are obtained from the different algorithms. Most algorithms do not consider whether miRNA genes and their potential target are co‐expressed and this could be an effective way of improving predictions. Furthermore both miRNAs and their targets are located in areas of the genome that are not often covered by whole exome sequencing. However, as larger scale WGS datasets become available, variation in 3’UTRs and intronic/intragenic regions will be better described, making it possible to begin to understand the functional role of these regions.

## 4 Conclusions

In summary, we report a single recurrent variant located close to in the miR‐708 gene that may have a role in susceptibility to BD and/or SCZ. This finding awaits replication in independent SCZ and BD cohorts, as does a functional analysis of the potential consequences of this variant.

## Disclosures

The authors of this paper do not have any commercial associations that might pose a conflict of interest in connection with this manuscript.
